# Preliminary Exploration of Epidemiologic and Hemodynamic Characteristics of Restrictive Filling Diastolic Dysfunction Based on Echocardiography in Critically Ill Patients: A Retrospective Study

**DOI:** 10.1155/2018/5429868

**Published:** 2018-02-21

**Authors:** Yi Li, Wanhong Yin, Yao Qin, Xueying Zeng, Tongjuan Zou, Xiaoting Wang, Yangong Chao, Lina Zhang, Yan Kang, Chinese Critical Ultrasound Study Group (CCUSG)

**Affiliations:** ^1^Department of Critical Care Medicine, West China Hospital of Sichuan University, 37 Guoxue Avenue, Chengdu 610041, China; ^2^Department of Critical Care Medicine, Peking Union Medical College Hospital, Peking Union Medical College, Chinese Academy of Medical Sciences, Beijing 100730, China; ^3^Department of Critical Care Medicine, The First Hospital of Tsinghua University, Beijing 100016, China; ^4^Department of Critical Care Medicine, Xiangya Hospital, Central South University, Changsha, Hunan 410008, China

## Abstract

**Objective:**

To preliminarily describe the epidemiologic and hemodynamic characteristics of critically ill patients with restrictive filling diastolic dysfunction based on echocardiography.

**Setting:**

A retrospective study.

**Methods:**

Epidemiologic characteristics of patients with restrictive filling diastolic dysfunction in ICU were described; clinical and hemodynamic data were preliminarily summarized and compared between patients with and without restrictive filling diastolic dysfunction; most of the data were based on echocardiography.

**Results:**

More than half of the patients in ICU had diastolic dysfunction and about 16% of them had restrictive filling pattern. The patients who had restrictive filling diastolic dysfunction were more likely to have wider diameter of IVC (2.18 ± 0.50 versus 1.92 ± 0.43, *P* = 0.037), higher extravascular lung water score (15.9 ± 9.2 versus 13.2 ± 9.1, *P* = 0.014), lower left ventricular ejection fraction (EF-S: 53.0 ± 16.3 versus 59.3 ± 12.5, *P* = 0.014), and lower percentage of normal LAP that was estimated by* E*/*e*′ (8.9% versus 90.0%, *P* = 0.001) when compared with those of patients without restrictive filling diastolic dysfunction.

**Conclusion:**

Our results suggest that critically ill patients with restrictive filling diastolic dysfunction may experience rising volume status, increasing extravascular lung water ultrasonic score, reducing long-axis systolic dysfunction, and less possibility of normal left atrial pressure. Intensivists are advised to pay more attention to patients with diastolic dysfunction, especially the exquisite fluid management of patients with restrictive filling pattern due to the close relationship of restrictive filling diastolic dysfunction with volume status and extravascular lung water in our study.

## 1. Introduction

Left ventricular diastolic dysfunction is quite common in critically ill patients [1–4] since there are so many predisposing factors existing, such as the complex medical history or underlying diseases (hypertension), acute conditions (sepsis, myocardial ischemia), and special therapies (volume loading, positive end-expiration pressure) in intensive care units. However, diastolic dysfunction had been greatly underestimated for a long time until the development of critical care echocardiography, which is portable, easy-to-use, and playing a vital role in identifying diastolic dysfunction at the bedside, recently. Since data [2, 4, 5] indicated that morbidity and mortality in patients with advanced diastolic heart failure are as poor as in patients with systolic heart failure, diastolic dysfunction in critically ill patients has aroused more and more attention of intensivists.

Diastolic dysfunction refers to the anomaly of the ability of the left ventricle to fill up; its normal function mainly depends on the active relaxation and passive compliance of left ventricular myocardium. Diastolic dysfunction, especially restrictive filling diastolic dysfunction, is likely to occur and exerts an adverse effect on critically ill patients. It has been demonstrated to have a strong association with weaning failure [6, 7], abrupt pulmonary edema [8, 9], and the outcome of sepsis [10–12] in ICU patients. However, Saleh and Vieillard-Baron [13] pointed out that they still had little data from large studies regarding the incidence, characteristics, and impact on prognosis of left ventricular diastolic dysfunction in ICU.

In this paper, we screened ultrasonic hemodynamic parameters from a critical care ultrasound database, which included the patients admitted to a general ICU for a whole year, and reviewed the electronic medical record to collect clinical data. We divided these patients into restrictive filling and nonrestrictive filling group. Here, for the first time, we preliminarily described the epidemiologic and hemodynamic characteristics of critically ill patients with restrictive filling diastolic dysfunction based on echocardiography.

## 2. Materials and Methods

### 2.1. Patients and Data Sources

This was a retrospective study that included all patients in a critical care ultrasound database that was built in a general critical care unit of an academic teaching hospital for a whole year from November 1, 2014, to October 31, 2015. All adult patients (not less than 18 years of age) were admitted in ICU during that period and were recruited in this database. The data of patients with obvious cardiac structural or valvular abnormalities or lack of parameters of identifying diastolic function, which include the peak mitral inflow *E* and *A* velocity waves on pulsed-Doppler and the diastolic* e*′ and* a*′ peak velocity on tissue Doppler, were excluded from our analysis.

Clinical assessment would be carried out immediately after they were admitted in ICU and critical care ultrasonic examinations were completed within 24 h after that. Critical care ultrasonic examinations, which included cardiac and lung ultrasound examination according to standard [14, 15], were completed by an Ultrasound Crew which consists of 6 intensivists who were well-trained in critical care ultrasound. A portable PHILIPS CX50 ultrasonic machine (CX 50 and Version 3.0, PHILLIPS Healthcare, Bothell, USA) was used to conduct this examination. This critical care ultrasonic examination evaluated the basic structural and valvular abnormality of heart, function of left and right ventricle, and volume status and calculated lung ultrasound pulmonary edema score. Board-certified cardiologists reviewed all the images to ensure the accuracy of all cardiac function parameters which were recorded on an excel sheet.

Restrictive filling diastolic dysfunction, the most serious pattern of diastolic dysfunction, is considered to have the most apparent impact on hemodynamics of patients. To explore this, we grouped our patients in restrictive filling diastolic dysfunction and nonrestrictive filling diastolic dysfunction according to the standards described in ASE/EACVI GUIDELINES [16] and compared their data of clinical and hemodynamics, which were reviewed from the electronic medical record and the critical care ultrasound database, respectively. The study was approved by the regional ethical review board in Chengdu, China. The data was obtained using a standard of care clinical protocol; the regional ethical review board waived the requirement of informed consent.

### 2.2. Measurements

We retrospectively reviewed the electronic medical record and the following data were recorded: age; gender; APACHE (Acute Physiology and Chronic Health Evaluation) II score; diagnosis; past medical history (hypertension, diabetes mellitus); heart rate, blood pressure and respiratory rate when performing critical care ultrasound examination; in-hospital mortality and 28-day mortality; and lengths of mechanical ventilation and staying in ICU; oxygenation index was calculated by dividing oxygen partial pressure, which was obtained through blood gas analysis performed within 24 h around critical care ultrasonic examination, by the corresponding oxygen concentration.

Parameters measured by critical care ultrasound, related to cardiac function, volume status, and pulmonary edema, were selected from the critical care ultrasound database. The peak mitral inflow *E* and *A* velocity waves on pulsed-Doppler and the systolic* s*′ and the diastolic* e*′ and* a*′ peak velocity on tissue Doppler imaging at the lateral mitral annulus were measured from the apical four-chamber view and the *E*/*A* ratio was calculated. From the same view, left ventricular ejection fraction (EF-s) was measured using Simpson's method for monoplane; EF-M was measured using M-mode in long-axis view; MAPSE was measured using M-mode and MV-SD was measured using tissue Doppler at the lateral mitral annulus from the apical four-chamber view. If tricuspid regurgitation existed, pressure gradient would be measured and recorded. Systolic pulmonary arterial pressure could be estimated by adding pressure gradient and right atrial pressure, which is estimated by diameter and variation of inferior vena cava.

The inferior vena cava diameter was identified in the subcostal long-axis view and measured from a frozen M-mode image at the hepatic segment of the IVC just cephalic to the origin of the hepatic vein or 2 cm away from conjunction of IVC and right atrium in end-expiratory phase. Left ventricular end-diastolic diameter and left atrial end-systolic diameter were measured inferior to the mitral leaflets from a parasternal left ventricle long-axis view. The value of lateral* E*/*e*′ < 8 is considered a normal pulmonary artery occlusion pressure (PAOP) and the left atrial pressure [17].

**Figure 1:**
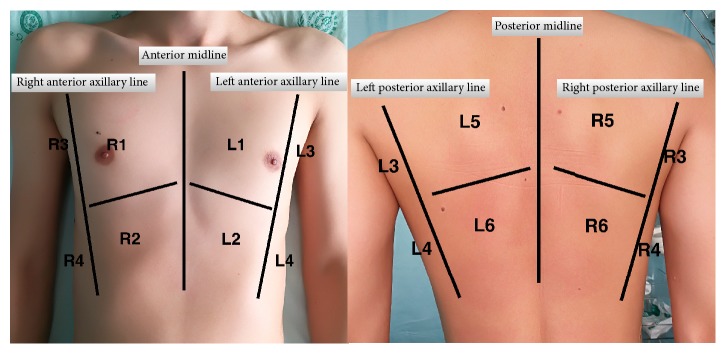
12 regions of chest wall. Each hemithorax was divided into 3 areas by anterior and posterior axillary line: anterior, lateral, and posterior. Each area of chest wall was divided into upper and lower halves. Therefore, 12 regions of chest wall are R1–R6 (right thorax) and L1–L6 (left thorax).

Ultrasonic extravascular lung water score was calculated by adding score of each area of chest wall. There are 12 regions of chest wall in total. Each hemithorax was divided into 3 areas by anterior and posterior axillary line: anterior, lateral, and posterior. Each area of chest wall was divided into upper and lower halves. Therefore, 12 regions of chest wall are R1–R6 (right thorax) and L1–L6 (left thorax) (Figure 1). A lines or fewer than two isolated B lines, multiple and well-defined B lines (B1 lines), multiple coalescent B lines (B2 lines), and lung consolidation (C) were given 0, 1, 2, and 3 points correspondingly. The worst sign of this area determined the score of each area [18].

### 2.3. Statistics

SPSS 20.0 was used for statistical analysis. Categorical variables are described as numbers (percentages) and continuous variables as mean (± standard deviation). Categorical variables were compared with chi-square test and continuous variables were compared with independent *t*-test. *P* value less than 0.05 was considered statistically significant.

## 3. Results

We included 451 patients who were admitted to a general critical care unit of an academic teaching hospital in China for a whole year from November 1, 2014, to October 31, 2015. Of those, 74 patients were excluded from the analysis as a result of at least one exclusion criterion. A total of 377 patients were analyzed.

### 3.1. Description of Demography and* Characteristics of Epidemiology*

#### 3.1.1. Characteristics of Demography

Demographic characteristics of population in this study were summarized in Table 1. The average age was 57 years. The youngest patient was 18 years old and the oldest was 97 years old. There were 203 (54%) patients who were between 18 and 60 years old and another 174 (46%) more than 60 years old. Approximately 40% of the patients were female and 60% were male. The average value of APACHE II, which was scored when the patients were admitted to ICU, was 19, the minimum was 2, and maximum was 45 (Table 1).

#### 3.1.2. Characteristics of Epidemiology

**Figure 2:**
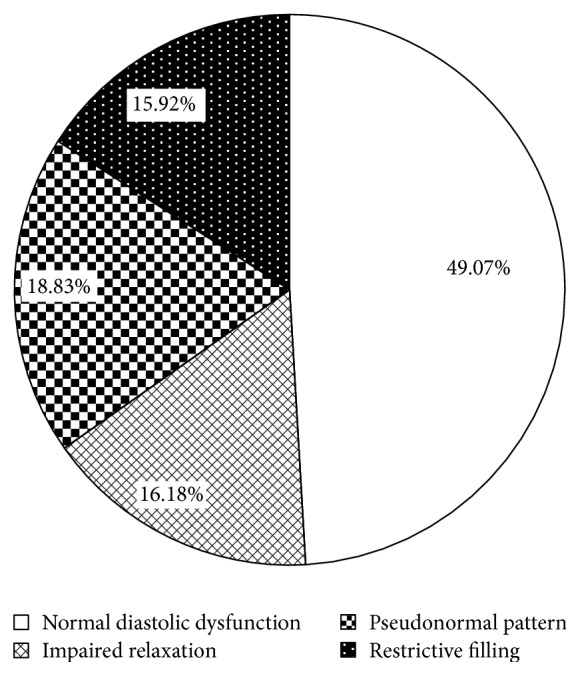
Distribution of different diastolic function degrees. Diastolic function was divided into normal diastolic dysfunction, impaired relaxation, pseudonormal pattern, and restrictive filling. More than half of patients in ICU had diastolic dysfunction and restrictive filling diastolic dysfunction was up to 15.92%.

**Figure 3:**
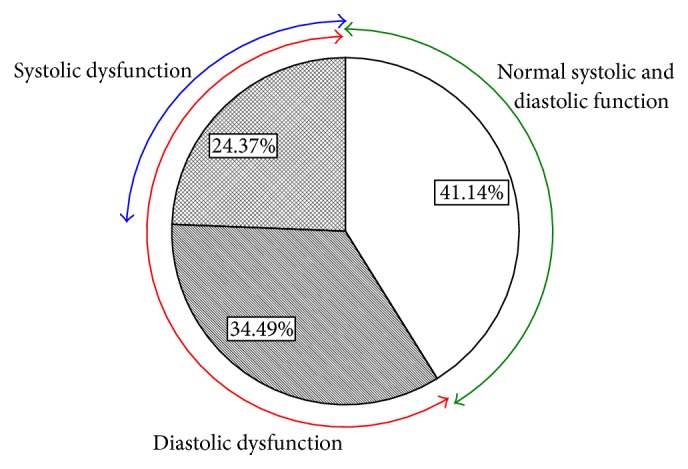
Distribution of different systolic and diastolic dysfunction. Empty space (also green arrow) denotes patients with normal systolic and diastolic function; stripes denote patients with only diastolic dysfunction; grid (also blue arrow) denotes patients with both systolic dysfunction and diastolic dysfunction; red arrow (also the strips and the grid) denotes patients with diastolic dysfunction.

Distribution of patients with different diastolic function degrees was shown in Figure 2. There are 49.07%, 16.18%, 18.83%, and 15.92% of the patients in normal diastolic function group, impaired relaxation group, pseudonormal pattern group, and restrictive filling group, respectively. Distribution of patients with different systolic and diastolic dysfunction was shown in Figure 3. We can see that there are 58.86% of the patients who had diastolic dysfunction and 24.37% who had both systolic and diastolic dysfunction.

### 3.2. Impact of Restrictive Filling Diastolic Dysfunction to Hemodynamics

#### 3.2.1. Baselines and Clinical Data

The proportion of male patients of nonrestrictive filling group was almost the same as that of restrictive filling group. But the patients of nonrestrictive filling group were significantly younger, and APACHE II was remarkably lower when compared with patients in restrictive filling group (Table 2). The values of heart rate, mean arterial pressure (MAP), respiratory rate (RR), oxygenation index, in-hospital mortality, 28-day mortality, lengths of mechanical ventilation, and lengths of staying in ICU are listed in Table 2. There were no differences of these parameters between groups.

#### 3.2.2. Volume Status

The values of LAESD, LVEDD, and IVC in restrictive filling group were significantly wider than those in nonrestrictive filling group (LAESD: 3.67 ± 1.43 versus 2.78 ± 0.73, *P* = 0.001; LVEDD: 4.52 ± 0.93 versus 4.16 ± 0.71, *P* = 0.039; IVC: 2.18 ± 0.50 versus 1.92 ± 0.43, *P* = 0.037).

#### 3.2.3. Left Ventricular Filling Pressure

The values of lateral* E*/*E*′ and percentages of increased pressure of PAOP were obviously higher in restrictive filling group when compared with nonrestrictive filling group (lateral* E*/*E*′: 15.64 ± 5.50 versus 7.71 ± 3.40, *P* < 0.001; elevation of PAOP: 90.0% versus 8.9%, *P* = 0.001)

#### 3.2.4. Systolic Function of Left Ventricle

The values of EF-S, MV-SD, and MAPSE in the patients of restrictive diastolic dysfunction group were all remarkably lower than those of nonrestrictive diastolic dysfunction group (EF-S: 53.0 ± 16.3 versus 59.3 ± 12.5, *P* = 0.014; MV-SD: 8.8 ± 3.2 versus 11.6 ± 4.0, *P* < 0.001; MAPSE: 1.20 ± 0.62 versus 1.43 ± 0.53, *P* = 0.006). However, the value of EF-M had no statistically significant differences between groups (EF-M: 57.87 ± 20.37 versus 62.31 ± 15.48, *P* = 0.089).

#### 3.2.5. Systolic Pulmonary Arterial Pressure

There were 54 patients that had tricuspid regurgitation: 44 patients belonged to nonrestrictive diastolic dysfunction group and 10 belonged to restrictive diastolic dysfunction group. Estimated systolic pulmonary arterial pressure was significantly higher in restrictive filling diastolic dysfunction group than that in nonrestrictive filling diastolic dysfunction group (46.6 ± 15.0 versus 36.9 ± 16.9, *P* = 0.008)

#### 3.2.6. Score of Extravascular Lung Water

The total score of extravascular lung water and the score of zone 1 plus zone 2 of restrictive diastolic dysfunction group were remarkably higher than those of nonrestrictive diastolic dysfunction group (extravascular lung water score: 15.9 ± 9.2 versus 13.2 ± 9.1, *P* = 0.040; extravascular lung water score (zone 1 + zone 2): 2.69 ± 3.09 versus 1.77 ± 2.47, *P* = 0.032). However, the score of extravascular lung water of the rest of lung except zone 1 and zone 2 had no statistically significant differences between groups (*P* = 0.089)

## 4. Discussions

Because the diastolic dysfunction has been realized to play an important role in weaning failure, abrupt pulmonary edema, and prognosis of sepsis in critically ill patients, it has caught the intensivists' intention. However, lacking data from large-sample studies regarding its epidemiology and impact on hemodynamics of patients in intensive care units is still a big problem [13]. We performed this retrospective research and tried to preliminarily describe the characteristics of epidemiology and its impact on hemodynamics of restrictive filling diastolic dysfunction in critically ill patients.

In our study, the epidemiologic investigation found that the incidence of diastolic dysfunction was high (58.86%) and percentage of restrictive filling diastolic dysfunction was about 16%. There are 49.07%, 16.18%, 18.83%, and 16.92% of ICU patients having normal diastolic function, impaired relaxation, pseudonormal pattern, and restrictive filling in our study, respectively. There are no related data of ICU patients in other studies. One community based analysis [19] showed 70.4% of the 3,571 African Americans had normal diastolic function and 18.0%, 10.6%, and 0.9% had impaired relaxation, pseudonormal pattern, and restrictive filling, respectively.

When the diastolic dysfunction progressed to restrictive filling pattern, the diameters of IVC, LAESD, and LVEDD were significantly increased. Since the diameters of IVC and LVEDD were indicators of volume status in critical care ultrasound, we could consider that patients with restrictive filling diastolic dysfunction experience also increase of volume status.

A normal LAP, which is represented by PAOP of no more than 18 mmHg, could be identified by* E*/*e*′ being no more than 8 [17]. The percentage of patients with normal LAP evaluated by* E*/*e*′ was remarkably lower in restrictive filling group when compared with nonrestrictive filling group, which indicated close relationship between restrictive filling diastolic dysfunction and left ventricular filling pressure. There are limits of noninvasive evaluation of central venous pressure or measurement of pulmonary capillary wedge pressure in our study and limits of using a single parameter to estimate LAP since guidelines [16] already created a complex method to evaluate LAP through echocardiography. However, method to evaluate LAP in the guideline was not especially aimed at ICU patients, while the parameter* E*/*e*′ < 8 estimating a normal LAP demonstrated good sensitivity and specificity in ICU ventilated patients and was more suitable for the patients in our study. Furthermore, the parameter* E*/*e*′ is simple to measure and is applied in clinical practice. Therefore, we used this single parameter* E*/*e*′ to estimate LAP and reflect its relationship with restrictive filling diastolic dysfunction in ICU patients.

MV-SD and MAPSE are good indicators being used to describe long-axis systolic function and EF-m, which is measured by M-mode, which can represent short-axis systolic function of left ventricle. Studies showed that, in patients with diastolic heart failure, there is left ventricular longitudinal systolic impairment [20, 21]. In our study, the global systolic function of LV was obviously reduced in patients with restrictive filling diastolic dysfunction, as well as both MV-SD and MAPSE. However, EF-M, which indicates short-axis systolic function of left ventricle, had no statistically significant differences whether the patients had restrictive filling diastolic dysfunction or not. Vinereanu et al.'s study [22] demonstrated that worsening global diastolic dysfunction of the left ventricle is associated with a progressive decline in longitudinal systolic function. These suggest that patients with restrictive filling diastolic dysfunction may manifest the left ventricular longitudinal systolic impairment and the long-axis systolic dysfunction might be the previous phase of the global systolic dysfunction [23].

Results showed that estimated systolic pulmonary arterial pressure in our study was significantly higher in restrictive filling diastolic dysfunction group. This indicated the close relationship between systolic pulmonary arterial pressure and restrictive filling diastolic dysfunction but it was hard to clarify the substantial cause and effect between them and further study was needed.

Many studies [8, 9] have shown patients with diastolic dysfunction were vulnerable to pulmonary edema. Our study also found that when patients had restrictive filling diastolic dysfunction, extravascular lung water score was observed to be obviously elevated, especially in anterior lung regions that more likely represent cardiogenic pulmonary edema. This reminds us of the restrictive filling diastolic dysfunction which may affect the condition of extravascular pulmonary edema.

In our study, there was no statistically significant difference of mortality whether the patients had restrictive filling diastolic dysfunction or not. This result seems to be a little contradictive to some other researches [2, 4, 5, 10–12]. There are three reasons that may be involved. Firstly, this is a retrospective study; lack of consistency of baselines between groups could impact clinical outcome. Secondly, our study is aimed at the impact of restrictive filling diastolic dysfunction on pathologic and physiologic hemodynamics and it is not a prospective study that is aimed at the outcome. Thirdly, the studies that showed higher morbidity of patients with diastolic dysfunction had different populations with diastolic heart failure [2, 4, 5] or sepsis [10–12]. With regard to the impact of diastolic dysfunction to morbidity, there is still evidence with contradiction [24, 25].

This study is the first large-sample investigation lasting for one year, regarding restrictive filling diastolic dysfunction in the population of patients in ICU. It provides us with background information related to restrictive filling diastolic dysfunction in critically ill patients, describes the impact and characteristics of restrictive filling diastolic dysfunction on hemodynamics and pulmonary edema, and provides us with details and reference for further study. We will conduct a cohort study to investigate the impact of different diastolic dysfunction degrees on clinical outcome.

## 5. Conclusions

In conclusion, our results suggest that critically ill patients with restrictive filling diastolic dysfunction may be accompanied with rising volume status, increasing extravascular lung water ultrasonic score of zone 1 and zone 2, reducing long-axis systolic dysfunction, and less possibility of normal left atrial pressure. With the prevalence of diastolic dysfunction in ICU, intensivists are advised to pay more attention to patients with it, especially the exquisite fluid management to patients with restrictive filling pattern due to the close relationship of restrictive filling diastolic dysfunction with volume status and extravascular lung water in our study.

## Figures and Tables

**Table 1:** Demographic characteristics of the study population.

Characteristic	Numbers (percentage)	Average (minimum, maximum)
Age (years)		57 (18, 97)
18–60	203 (54%)	
>60	174 (46%)	
Gender		
Female	148 (39%)	
Male	229 (61%)	
APACHE II score		19 (2, 45)

**Table 2:** Baselines and clinical data between restrictive and nonrestrictive filling group.

	Nonrestrictive filling group (*n* = 317)	Restrictive filling group (*n* = 60)	*P* value
Age (yrs)	56.0 ± 18.7	61.3 ± 16.7	0.042^*∗*^
Gender: male (%)	195 (61.5)	34 (56.7)	0.481
APACHE II	18.6 ± 7.8	21.0 ± 8.2	0.033^*∗*^
Heart rate	95.7 ± 21.7	93.3 ± 23.4	0.441
Mean arterial pressure	85.7 ± 12.6	84.6 ± 15.8	0.600
Respiratory rate	18.5 ± 5.1	17.7 ± 4.3	0.298
Oxygenation index	227.8 ± 124.8	216.4 ± 145.6	0.534
Length of staying in ICU	15.3 (5, 20)	14.6 (6, 23)	0.736
Length of mechanical ventilation	184.9 (47, 329)	197.0 (57, 265)	0.661
In-hospital mortality (%)	29.3	35.0	0.381
28-day mortality (%)	26.7	36.7	0.115
Interior vena cava diameter	1.92 ± 0.43	2.18 ± 0.50	0.037^*∗*^
Left ventricular end-diastolic diameter	4.16 ± 0.71	4.52 ± 0.93	0.039^*∗*^
Enlargement of LV (%)	7.0	18.6	0.004^*∗*^
Left atrial end-systolic diameter	2.78 ± 0.73	3.67 ± 1.43	0.001^*∗*^
Enlargement of LA (%)	3.8	25.4	<0.001^*∗*^
Lateral *E*/*E*′	7.71 ± 3.40	15.64 ± 5.50	<0.001^*∗*^
Elevation of PAOP (%)	8.9	90.0	<0.001^*∗*^
Ejection fraction-S	59.3 ± 12.5	53.0 ± 16.3	0.014^*∗*^
Ejection fraction-M	62.31 ± 15.48	57.87 ± 20.37	0.189
MV-SD	11.6 ± 4.0	8.8 ± 3.2	<0.001^*∗*^
MAPSE	1.43 ± 0.53	1.20 ± 0.62	0.006^*∗*^
TV-SD	15.80 ± 4.85	14.38 ± 4.14	0.056
TAPSE	1.95 ± 0.56	1.77 ± 0.61	0.051^*∗*^
Extravascular lung water score	13.2 ± 9.1	15.9 ± 9.2	0.040^*∗*^
Extravascular lung water score (zones 1-2)	1.77 ± 2.47	2.69 ± 3.09	0.032^*∗*^
Extravascular lung water score (zones 3–6)	11.46 ± 7.89	13.20 ± 6.99	0.089

^*∗*^ *P* < 0.05.
